# Analysis of Kerr Noise in Angular-Rate Sensing Based on Mode Splitting in a Whispering-Gallery-Mode Microresonator

**DOI:** 10.3390/mi10020150

**Published:** 2019-02-23

**Authors:** Zhaohua Yang, Dan Li, Yuzhe Sun

**Affiliations:** School of Instrumentation Science and Opto-electronics Engineering, Beihang University, Beijing 100083, China; leedan05@foxmail.com (D.L.); sunyuzhe@buaa.edu.cn (Y.S.)

**Keywords:** mode splitting, Kerr noise, angular-rate sensing, whispering-gallery-mode, optical microresonator

## Abstract

Whispering-gallery-mode (WGM) microresonators have shown their potential in high-precision gyroscopes because of their small volume and high-quality factors. However, Kerr noise can always be the limit of accuracy. Angular-rate sensing based on mode splitting treats backscattering as a measured signal, which can induce mode splitting, while it is considered as a main source of noise in conventional resonator optical gyroscopes. Meanwhile, mode splitting also provides superior noise suppression owing to its self-reference scheme. Kerr noise in this scheme has not been defined and solved yet. Here, the mechanism of the Kerr noise in the measurement is analyzed and the mathematical expressions are derived, indicating the relationship between the Kerr noise and the output of the system. The influence caused by Kerr noise on the output is simulated and discussed. Simulations show that the deviation of the splitting caused by Kerr noise is 1.913 × 10^−5^ Hz at an angular rate of 5 × 10^6^ °/s and the corresponding deviation of the angular rate is 9.26 × 10^−9^ °/s. It has been proven that angular-rate sensing based on mode splitting offers good suppression of Kerr noise.

## 1. Introduction

In a whispering-gallery-mode (WGM) microresonator, the light wave is strongly confined in time and space by continuous total reflection, also described as a high Q factor and a small mode volume, respectively [1]. The light-matter interaction is enhanced, which makes the WGM microresonator extremely sensitive to weak signals, thereby making it useful for ultrasensitive detection [1,2]. It draws much attention to the field of sensors, including bio sensing, temperature sensing, pressure sensing, displacement sensing and gas sensing [3,4,5,6,7,8,9,10,11,12]. Modal coupling does not exist in the ideal WGM microresonator, where two WGMs (clockwise: CW and counterclockwise: CCW) propagate with a degenerate resonant frequency. While the degeneracy resonance will be split into two non-degenerate standing-wave modes (the symmetric mode (SM) and asymmetric mode (ASM)) if the symmetric resonator experiences any perturbation in the field distributions, such as structure defects, scattering around the cavity or rotation, namely, mode splitting [13,14,15]. Because of the successful demonstration in theory and experiments of nanoparticle detection by the above mode splitting, a new scheme for sensing has been developed [16,17,18,19].

Rotation of a resonator can also redistribute the internal modes in the frequency domain and give rise to mode splitting [18]. By detecting the frequency difference between the SM and ASM in the angular rate sensing based on mode splitting, good noise suppression is offered because of the same cavity environment experienced by the two modes. It was confirmed that in conventional optical gyroscopes, as a kind of non-reciprocal noise, Kerr noise is a kind of main sources of system bias [20]. The optical Kerr effect is a third-order nonlinear effect, characterized by a change in the refractive index of the material in response to a light field, intuitively, with field intensity. As early as 1982, S. Ezekiel et al. observed a non-reciprocal bias in the interferometric fiber gyroscope, caused by the difference in power of two waves propagating in the opposite directions [21]. In conventional resonant optical gyroscopes, the output bias owing to Kerr noise is always proportional to the intensity mismatch [20,22]. The non-reciprocal noise of the optical path structure in the angular rate sensing based on mode splitting has been reported before [23]. However, Kerr noise has not been analyzed.

In this paper, the analysis of Kerr noise in angular rate sensing based on mode splitting in a WGM optical microresonator is developed, a new idea of introducing Kerr noise into angular-rate measurement is proposed and a mathematical model is built after the mechanism of the noise is analyzed theoretically. Simulations are also performed, showing that the deviation of splitting caused by Kerr noise is 1.913 × 10^−5^ Hz at an angular rate of 5 × 10^6^ °/s; the corresponding deviation of angular rate is 9.26 × 10^−9^ °/s, which demonstrates that a good suppression of the Kerr noise is provided.

## 2. Principle and Theoretical Model

### 2.1. Theoretical Model of Angular-Rate Sensing Based on Mode Splitting

**Figure 1:**
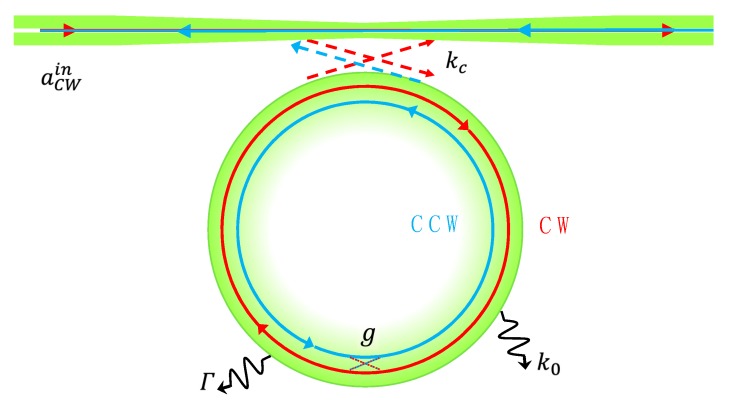
Sketch of the fiber-resonator coupled system, where $k_{0}$ is the intrinsic damping rate, $k_{c}$ is the microresonator-taper coupling rate, Γ is the additional damping rate because of scattering loss and g is the coupling coefficient of the light scattered into the resonator. $a_{CW}^{in}$ is the clockwise (CW) input field in the fiber taper.

Backscattering is created, resulting in mode splitting owing to inevitable imperfections of the resonator itself, such as structural defects or surface contaminations, which are called intrinsic splitting and should be regarded as zero bias. However, the amount of intrinsic splitting is small, always less than 2/10^7^ of the laser frequency and difficult to measure accurately. Thus, a subwavelength particle is introduced into the resonator as Rayleigh scatter to induce splitting, which is considered as static splitting together with intrinsic splitting. The fiber-resonator coupled system is shown in Figure 1.

Different splitting amounts can be obtained when the resonator rotates at disparate angular rates so that a correspondence can be established. Given a circular resonator with a radius *R* and a CCW rotation rate Ω, the frequency deviation of CW (CCW) per unit time is written as:

(1) $$\Delta \omega_{u}=\frac{1}{2}\frac{\Delta \varphi }{\tau_{r}}=\frac{\omega_{c}R}{n_{\mathrm{eff}}c}\Omega .$$

Here, $\Delta \varphi =8\pi^{2}R^{2}\Omega /\lambda c$ denotes the phase difference between CW and CCW per round owing to the rotation of the resonator based on the Sagnac effect. $\tau_{r}=2\pi Rn_{\mathrm{eff}}/c$ is the time that light takes traveling one round in the resonator. c is the speed of light in vacuum, $n_{\mathrm{eff}}$ is the effective refracting. The rate equations of the coupled fiber-microresonator system in the presence of a particle with allowance for rotation can be expressed as [18]:

(2) $$\frac{d}{dt}\left[\begin{array}{c} a_{\mathrm{CW}}\\ a_{\mathrm{CCW}} \end{array}\right]=\left[\begin{array}{cc} -\left(i\left(\omega_{c}+g+\Delta \omega_{u}\right)+\frac{k_{\mathrm{eff}}}{2}\right) & -\left(ig+\frac{\Gamma }{2}\right)\\ -\left(ig+\frac{\Gamma }{2}\right) & -\left(i\left(\omega_{c}+g-\Delta \omega_{u}\right)+\frac{k_{\mathrm{eff}}}{2}\right) \end{array}\right]\left[\begin{array}{c} a_{\mathrm{CW}}\\ a_{\mathrm{CCW}} \end{array}\right]-\left[\begin{array}{c} \sqrt{K_{c}}\\ 0 \end{array}\right]a_{\mathrm{CW}}^{\mathrm{in}}$$

Here $a_{\mathrm{cw}}$ and $a_{\mathrm{ccw}}$ are the amplitudes of CW and CCW, $k_{\mathrm{eff}}$ is defined as the effective damping rate of the system. $k_{\mathrm{eff}}=\Gamma +k_{0}+k_{c}$, where $k_{0}$ is the intrinsic damping rate, $k_{c}$ is the microresonator-taper coupling rate, Γ is the additional damping rate owing to scattering loss, g is the coupling coefficient of the light scattered in the resonator. $a_{\mathrm{CW}}^{\mathrm{in}}$ is the CW input field in the fiber taper. 

The imaginary part of the eigenvalue obtained by the state-space method represents the resonant frequency. By transforming the system matrix (2) into a diagonal matrix, the resonant frequencies of SM and ASM can be derived as:

(3) $$\omega_{\mathrm{SM}}=\omega_{c}+g+\frac{\Gamma g}{\sqrt{2\left[\frac{\Gamma^{2}}{4}-g^{2}-\Delta {\omega_{u}}^{2}+\sqrt{{\left(\frac{\Gamma^{2}}{4}-g^{2}-\Delta {\omega_{u}}^{2}\right)}^{2}+\Gamma^{2}g^{2}}\right]}},$$

(4) $$\omega_{\mathrm{ASM}}=\omega_{c}+g-\frac{\Gamma g}{\sqrt{2\left[\frac{\Gamma^{2}}{4}-g^{2}-\Delta {\omega_{u}}^{2}+\sqrt{{\left(\frac{\Gamma^{2}}{4}-g^{2}-\Delta {\omega_{u}}^{2}\right)}^{2}+\Gamma^{2}g^{2}}\right]}}.$$

After simplification using $\delta_{0}=2g$, where $\delta_{0}$ represents the value of static splitting, it is straightforward that the amount of splitting can be calculated from $\delta =\left|\omega_{\mathrm{SM}}-\omega_{\mathrm{ASM}}\right|$ using Equations (3) and (4) as

(5) $$\delta =\sqrt{\delta_{0}^{2}+\frac{\Omega^{2}{\left(\frac{2\omega_{c}R}{n_{\mathrm{eff}}c}\right)}^{2}}{\left(1+\frac{\Gamma^{2}}{\delta^{2}}\right)}}.$$

Additional damping owing to scattering loss can be ignored when the resonator is in the absorption-limited regime and the relationship between the splitting value [18] and the angular rate can be found as

(6) $$\delta =2\sqrt{g^{2}+\Delta {\omega_{t}}^{2}}=\sqrt{\delta_{0}^{2}+\Omega^{2}{\left(\frac{2\omega_{c}R}{n_{\mathrm{eff}}c}\right)}^{2}},$$

also written as $\Omega =\frac{n_{\mathrm{eff}}c}{2\omega_{c}R}\sqrt{\delta^{2}-\delta_{0}^{2}}$.

### 2.2. Kerr Effect-Induced Noise Model

It is generally accepted that, owing to the difference in the intensities of CW and CCW, the optical Kerr effect as a nonlinear optical effect can induce an offset in the output of a conventional gyroscope system, which can lead to a gross error in a high-precision navigation system. The refractive index of a silica optical microresonator will change under the action of an electric and magnetic field, as well as in the case of a strong light field. In the scheme of angular rate sensing based on mode splitting, the light field density at the edge of the cavity is high because of the strong confinement of the WGM microresonator, when CW and CCW travel concurrently because of scattering. However, the intensities are always different and will lead to a change in the refractive indices, which will subsequently affect the propagation of resonant waves; thus, the Kerr effect-induced noise may also be one of the causes of non-reciprocity error in our scheme.

The Kerr effect in the path of CW (CCW) can be expressed as

(7) $$n_{\mathrm{CW}\left(\mathrm{CCW}\right)}=n_{1}+\gamma n_{2}I_{\mathrm{CW}\left(\mathrm{CCW}\right)}/S$$

where $n_{\mathrm{cw}\left(\mathrm{ccw}\right)}$ is the refractive index of CW (CCW), $n_{1}$ and $n_{2}$ denote, respectively, the normal refractive index and nonlinear refractive index coefficient of the cavity, $I_{\mathrm{CW}\left(\mathrm{CCW}\right)}$ represents the field power of CW (CCW), γ is the polarization factor and γ=1 is adopted here. S is the effective area of light concentration. In our scheme, the Kerr effect is introduced into the angular-rate sensing model and the time of CW and CCW light circling in the cavity is corrected as

(8) $$\tau_{\mathrm{cw}\left(\mathrm{ccw}\right)}=\frac{2\pi Rn_{\mathrm{cw}\left(\mathrm{ccw}\right)}}{c}=\frac{2\pi R\left(n_{1}+\gamma n_{2}I_{\mathrm{cw}\left(\mathrm{ccw}\right)}/S\right)}{c}.$$

It is clear that a time variation will appear owing to the imbalance of the field power. The frequency deviation of CW (CCW) per unit of time is given by

(9) $$\begin{array}{c} \Delta \omega_{\mathrm{tk}}=\frac{1}{2}\left(\frac{\Delta \varphi }{2\tau_{\mathrm{cw}}}+\frac{\Delta \varphi }{2\tau_{\mathrm{ccw}}}\right)=\frac{\frac{8\pi^{2}R^{2}}{\lambda c}\Omega }{\frac{4\times 2\pi Rn_{\mathrm{cw}}}{c}}+\frac{\frac{8\pi^{2}R^{2}}{\lambda c}\Omega }{\frac{4\times 2\pi Rn_{\mathrm{ccw}}}{c}}=\frac{\omega_{c}R\Omega }{2c}\left(\frac{n_{\mathrm{cw}}+n_{\mathrm{ccw}}}{n_{\mathrm{cw}}n_{\mathrm{ccw}}}\right)=\\ \frac{\omega_{c}R\Omega }{2c}\left[\frac{2n_{1}+\gamma n_{2}\left(I_{\mathrm{cw}}+I_{\mathrm{ccw}}\right)/S}{\left(n_{1}+\gamma n_{2}I_{\mathrm{cw}}/S\right)\left(n_{1}+\gamma n_{2}I_{\mathrm{ccw}}/S\right)}\right]. \end{array}$$

Considering Kerr noise with respect to δ, the splitting amount $\delta_{k}$ becomes

(10) $$\delta_{k}=\sqrt{\delta_{0}^{2}+\Omega^{2}{\left\{\frac{\omega_{c}R}{2c}\left[\frac{2n_{1}+\gamma n_{2}\left(I_{\mathrm{cw}}+I_{\mathrm{ccw}}\right)/S}{\left(n_{1}+\gamma n_{2}I_{\mathrm{cw}}/S\right)\left(n_{1}+\gamma n_{2}I_{\mathrm{ccw}}/S\right)}\right]\right\}}^{2}}.$$

## 3. Simulation and Discussion

**Figure 2:**
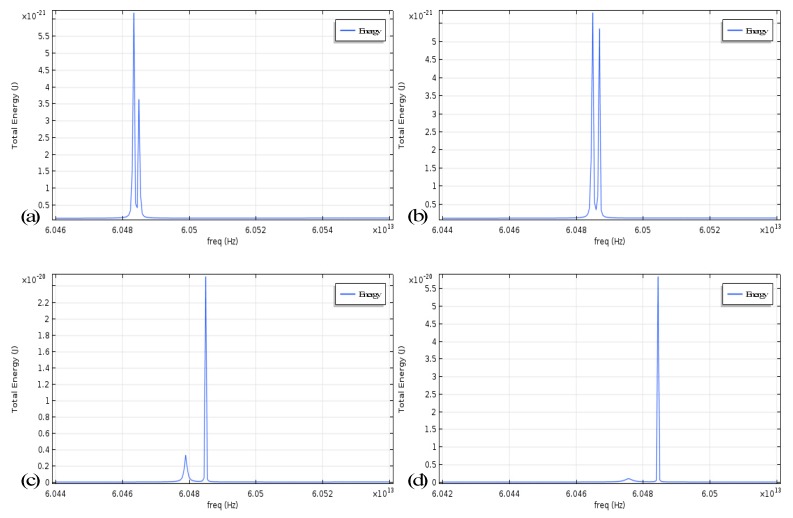
Light distribution of symmetric mode (SM) and asymmetric mode (ASM) in the cavity with mode splitting induced by different scattering: (**a**,**c**) Diameter 0.1 μm and 0.2 μm, tangent to the cavity; (**b**,**d**) Diameter 0.1 μm and 0.2 μm with a position of 0.05 μm further from the cavity than (**a**,**c**).

It is observed that the influence of Kerr noise on the system of angular-rate sensing based on mode splitting is owing to three factors: the angular rate of the microresonator, the light power distribution in the CW and CCW modes and the size of the cavity. The input laser power introduced for the evanescent field coupling to the left port of the fiber taper is usually fixed at one of the several commonly used values. It is not surprising that when we change the position and size of a particle relative to the cavity, the simulation result indicates that the power distribution of CW and CCW modes is greatly affected by scattering, as shown in Figure 2. Meanwhile, the size of the cavity also indirectly affects the effective cross-sectional area of the light concentration causing a slight change. 

**Figure 3:**
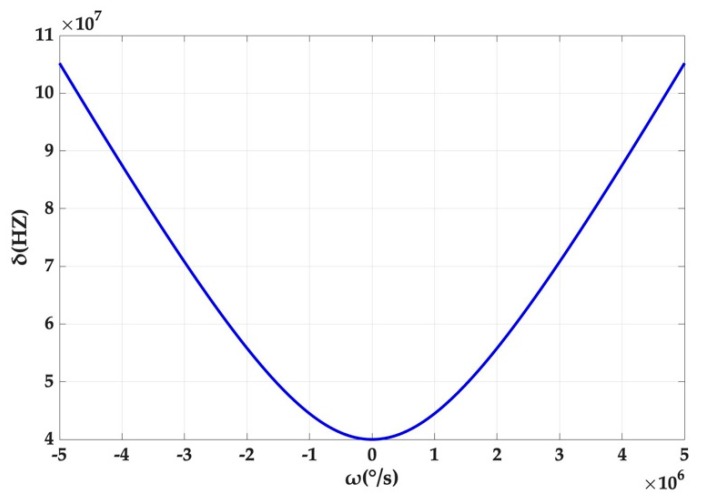
The contrast between the splitting value of the original model and the new model with Kerr noise introduced at different angular rates.

We compare the magnitude of the splitting of the original angular-rate measurement model with the case of taking into account Kerr noise, imparting different rotation rates to the cavity. The parameters are set as the conventional settings in the experiment, specific for $\lambda =780\;\mathrm{nm},R=100\;\mu m,\;n_{0}=1.445,\;p_{\mathrm{cw}}=2\;\mathrm{mw},\;\;p_{\mathrm{ccw}}=8\;\mathrm{mw}$. As the values of the two splitting values are extremely close to each other, the two curves in the Figure 3 almost completely coincide, making it difficult to observe the differences, even if they are locally increased. It can be preliminarily concluded that Kerr noise there does not have a strong effect. To circumvent this problem, we extract the difference in the splitting values in two cases, which can be understood as a measurement error (system offset) caused by Kerr noise. 

**Figure 4:**
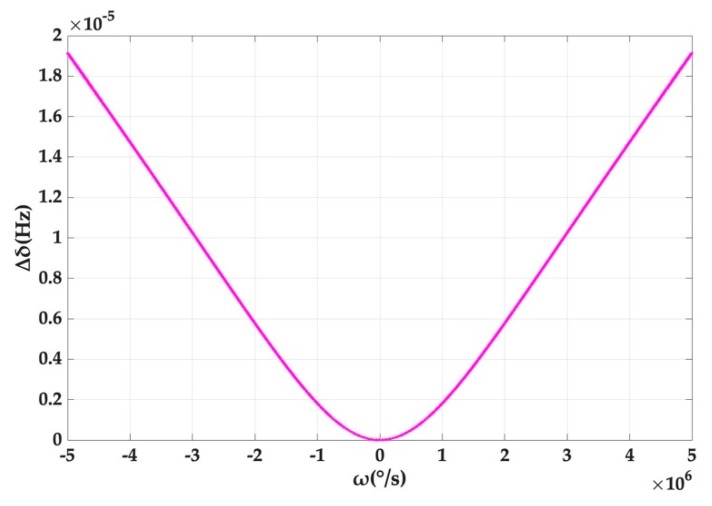
Splitting value deviation caused by Kerr noise at different angular rates.

As can be seen from Figure 4, the offset of the system output, induced by Kerr noise, increases with the rotation rate of the cavity and the gradient increases and approximately approaches a constant. However, it should be pointed out that the offset reaches only 1.913 × 10^−5^ Hz, even if the rotation rate is up to 5 × 10^6^ °/s, which corresponds to the splitting amount of 1.05 × 10^8^ Hz in Figure 3. This is also consistent with the previous result that the offset caused by Kerr noise is small.

**Figure 5:**
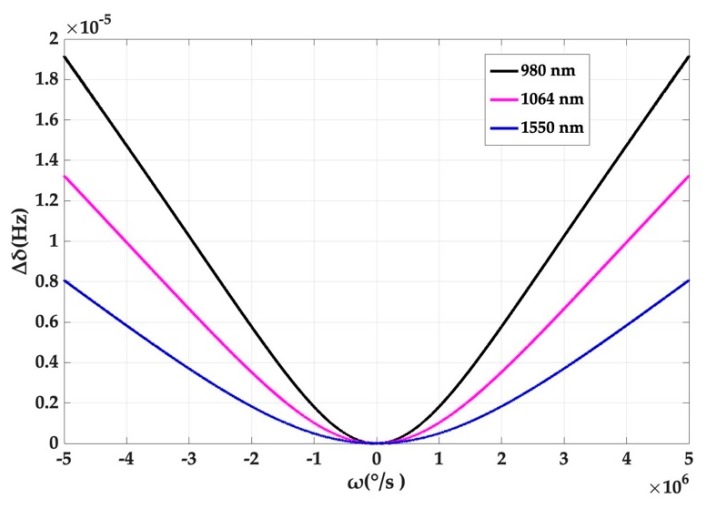
The relationship between the splitting value deviation and the angular rate at different wavelengths.

The relationship between the Kerr noise and the rotation angular rate of the resonator at 780 nm, 1064 nm and 1550 nm is also given in Figure 5. The output offset caused by Kerr noise decreases with increasing wavelength, providing guidance for choosing a longer wavelength of 1550 nm for sensing. However, the deviation is also small.

**Figure 6:**
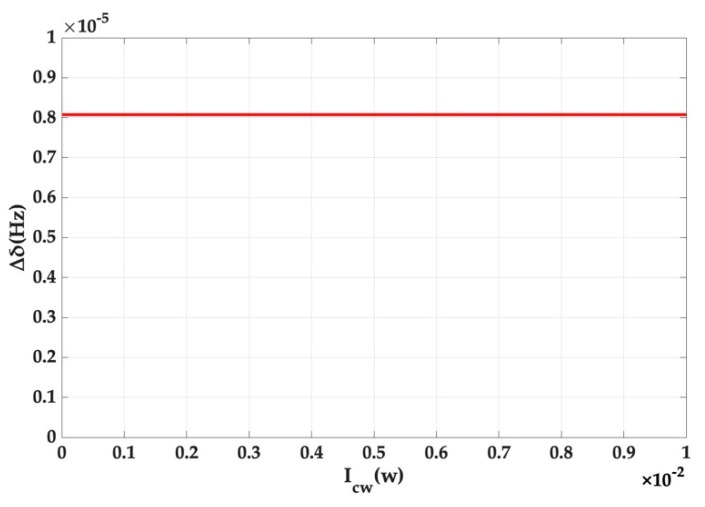
The relationship between the splitting value deviation and the angular rate for different distributions of light power.

In conventional gyroscopes, the imbalance of the power in two directions is responsible for the strong nonlinear Kerr effect. Here we have taken all possible distribution ratios of the light power at a rotation rate of 5 × 10^6^ °/s to better demonstrate the Kerr noise error in Figure 6.

The result is interesting but predictable. A small splitting value deviation caused by Kerr noise itself results in the insignificant change caused by the difference between the CW and CCW light power distributions and the image is shown as a horizontal line. It has been proven once again that the angular rate measurement system, based on mode splitting, well suppresses Kerr noise. All the simulation results presented above can reveal that the influence of Kerr noise on the output is weak.

## 4. Conclusions

In this paper, Kerr noise analysis in angular-rate sensing based on mode splitting in a WGM microresonator is performed. The mechanism of Kerr noise in the system was first analyzed. Subsequently, a theoretical model was constructed that considers Kerr noise while also providing a new idea on how to incorporate Kerr noise into a variety of angular-rate sensing models. Several simulations are carried out to visualize the influence on the output offset caused by Kerr noise. The deviation of splitting caused by Kerr noise is only 1.913 × 10^−5^ Hz at an angular rate of 5 × 10^6^ °/s, the corresponding deviation of the angular rate is 9.26 × 10^−9^ °/s, which is a slight impact on the offset. Relevant parameters, such as wavelength and light power distribution, are also discussed.

Taken together with our previous analysis, we can conclude that the offset caused by Kerr noise is very small in our system of angular-rate sensing based on mode splitting in a WGM microresonator, indicating that the sensing scheme is more immune to Kerr noise. The results offer a great support to the good characteristic of the microcavity angular-rate sensing based on mode splitting and show a wide prospect of application using a WGM optical microresonator as the core component.
